# Regulation of quorum sensing activities by the stringent response gene *rsh* in sphingomonads is species-specific and culture condition dependent

**DOI:** 10.3389/fmicb.2024.1368499

**Published:** 2024-04-04

**Authors:** Yue Xiao, Xin Chen, Hang Lu, Tingting Jiang, Yichun Wang, Luyi Liang, Sergey Dobretsov, Yili Huang

**Affiliations:** ^1^Zhejiang Provincial Key Laboratory of Organic Pollution Process and Control, Department of Environmental Science, College of Environmental and Resource Sciences, Zhejiang University, Hangzhou, China; ^2^National Demonstration Center for Experimental Environment and Resources Education, Zhejiang University, Hangzhou, China; ^3^UNESCO Chair, Department of Marine Science and Fisheries, College of Agricultural and Marine Sciences, Sultan Qaboos University, Muscat, Oman

**Keywords:** quorum sensing, stringent response, sphingomonads, *rsh*, acyl homoserine lactone, *luxI* homolog

## Abstract

Stringent response and quorum sensing (QS) are two essential mechanisms that control bacterial global metabolism for better survival. Sphingomonads are a clade of bacteria that survive successfully in diverse ecosystems. *In silico* survey indicated that 36 out of 79 investigated sphingomonads strains contained more than one *luxI* homolog, the gene responsible for the biosynthesis of QS signal acyl homoserine lactones (AHLs). Investigation of the regulatory effects of the stringent response gene *rsh* on QS related bioactivities were carried out using *rsh* mutants of *Sphingobium japonicum* UT26 and *Sphingobium* sp. SYK-6, both had three *luxI* homologs. Results indicated that deletion of *rsh* upregulated the overall production of AHLs and extracellular polymeric substances (EPS) in both UT26 and SYK-6 in rich medium, but affected expressions of these *luxI/luxR* homologs in different ways. In the poor medium (1% LB), *rsh* mutant of SYK-6 significantly lost AHLs production in broth cultivation but not in biofilm cultivation. The regulatory effects of *rsh* on QS activities were growth phase dependent in UT26 and culture condition dependent in SYK-6. Our results demonstrated the negative regulatory effect of *rsh* on QS activities in sphingomonads, which were very different from the positive effect found in sphingomonads containing only one *luxI/R* circuit. This study extends the current knowledge on the intricate networks between stringent response and QS system in sphingomonads, which would help to understand their survival advantage.

## 1 Introduction

As one of the most successful organisms in this world, bacteria employ sophisticated mechanisms to regulate their growth and metabolisms. Among these regulatory mechanisms, stringent response and quorum sensing (QS) are two essential mechanisms that control bacterial global metabolism (Waters and Bassler, 2005; Gaca et al., 2012).

Stringent response is a strategy used by bacteria to cope with environmental stress such as nutrients limitation, oxidative stress, etc. (Poole, 2012; Lu et al., 2023). The stringent response is mediated by guanosine tetraphosphate and guanosine pentaphosphate [(p)ppGpp], which is synthesized and hydrolyzed by members of the RSH superfamily (Dalebroux and Swanson, 2012). The corresponding stringent response gene *rsh* was reported to regulate various bacterial bioactivities including QS (Schafhauser et al., 2014; Lu et al., 2023).

The QS is a mechanism of cell-to-cell signaling involving the production of hormone-like signal molecules called autoinducers (Papenfort and Bassler, 2016). In Gram-negative bacteria, the QS signals are usually acyl-homoserine lactones (AHLs), which are produced by LuxI-type synthases (Fuqua et al., 2001). It is proved that QS involves in lots of activities that are crucial for bacterial survival, such as biofilm formation, motility, EPS production etc. (Sperandio et al., 2001; Tan et al., 2014).

Both stringent response and QS are favorable mechanisms for bacterial survival. A fundamental question is that how does stringent response interact with the QS system. Previous studies on *Pseudomonas* spp. and *Novosphingobium* spp. indicated that stringent response did interfere with QS systems in multiple ways (Gan et al., 2009; Schafhauser et al., 2014; Wang et al., 2022). Deletion of *spoT*, one of the stringent response gene, resulted in decreased AHLs production in *Pseudomonas protegens* SN15-2 (Wang et al., 2022). Whereas in *Pseudomonas aeruginosa*, (p)ppGpp-null mutant has reduced butanoyl-homoserine lactone (C4-HSL) and 3-oxo-dodecanoyl-homoserine lactone (3-oxo-C12-HSL) levels, but has increased biosynthesis of the other kind of QS signal, HHQ (4-hydroxyl-2-heptyl quinoline) and PQS (3,4-dihydroxy-2-heptylquinoline) (Schafhauser et al., 2014). The intricate network between stringent response and QS remains to be elucidated.

Sphingomonads, mainly consists of *Novosphingobium*, *Sphingopyxis*, *Sphingobium*, and *Sphingomonas*, attract great research interest due to their ubiquitous distribution and diverse metabolism capacity (Stolz, 2009; Glaeser and Kampfer, 2014; Huang et al., 2017; Sheng et al., 2022; Lu et al., 2023). Their sophisticated QS systems, often containing multiple *luxI/R* genes, might contribute to their survival advantage (Gan et al., 2015). In *Novosphingobium* sp. Rr 2–17, and *Novosphingobium pentaromativorans* US6-1, the *rsh* gene was necessary for the accumulation of AHLs, suggesting that QS was strictly under the regulation of *rsh* (Gan et al., 2009; Lu and Huang, 2018). Given that both US6-1 and Rr 2–17 have only one pair of *luxI/R* homologs in their genome, it is still preliminary to conclude that stringent response positively regulates the QS activities in sphingomonads. Strains with multiple *luxI/R* homologs in their genomes should be tested for the interaction between these two systems.

In this study, we surveyed the position and diversity of *luxI* homologs in sphingomonads. Then we chose *Sphingobium japonicum* UT26 and *Sphingobium* sp. SYK-6, which had three *luxI* homologs, to investigate the effect of *rsh* deletion on the QS-related bioactivities, such as AHL production, *luxI/R* gene expression and EPS production. Our results indicated that the regulatory effects of *rsh* on the QS related activities were species specific and culture condition dependent, which extended our understanding of the networks between QS and stringent response.

## 2 Materials and methods

### 2.1 Gene sequence survey and phylogenetic tree construction

All AHL synthase genes, *luxI/R* homologs, and *rsh* gene sequences were retrieved from NCBI Genome Database (Microbial Genomes www.ncbi.nih.gov) using the keywords “AHL synth(et)ase,” “quorum sensing,” “*luxI*” and “autoinducer.” Detailed information for these resulting genes such as sequences and gene locations were manually checked, and recorded for further analysis.

The amino acid sequences of these *luxI* homologs were aligned by clustalW using MEGA software (version 11), and the phylogenetic tree was constructed by the neighbor-joining method with 1,000 bootstrap.

### 2.2 Bacterial strains, plasmids and culture conditions

Bacterial strains and plasmids used in this study are listed in Table 1. *Sphingobium japonicum* UT26 and *Sphingobium* sp. SYK-6 were grown at 28°C in 1/3 LB media and LB media, respectively. Their *rsh* deletion mutants were grown in respective medium plus 100 μg/mL streptomycin. The AHL reporter strain *Agrobacterium tumefaciens* A136 and all *Escherichia coli* strains were grown at 37°C in LB medium with proper antibiotics (Lu and Huang, 2018).

**Table 1:** Strains and plasmids used in this study.

Strains or plasmids	Relevant characteristics	Source
Strains		
*Agrobacterium tumefaciens* A136(pCF218)(pCF372)	traI-lacZ fusion; AHL reporter	Laboratory collection
*E. coli* DH5a	F- hsdR17 endA1 thi-1 gyrA96 relA1 supE44 ΔlacU169 (φ80dlacZΔM15)	TransGen Biotech (Beijing, China)
*E. coli* BL21(DE3)	F- ompT hsdSB (rB- mB-) gal dcm (DE3)	TransGen Biotech (Beijing, China)
*E. coli* S17-1(λpir)	*E. coli* K-12 Tpr Smr recA thi hsdRM+ RP4::2-Tc::Mu::Km Tn7, λpir phage lysogen	Laboratory collection
*Sphingobium* sp. SYK-6	Wild type	Japan Collection of Microorganisms (JCM)
*Sphingobium japonicum* UT26	Wild type	Japan Collection of Microorganisms (JCM)
△*rsh*_SYK-6_	*rsh* deletion mutant of SYK-6	Constructed in this study
△*rsh*_UT26_	*rsh* deletion mutant of UT26	Constructed in this study
Plasmid		
pAK405	Suicide vector used for in-frame gene deletion	Addgene
pMD19-T	TA cloning vector	TaKaRa
pCM62	Broad-host-range cloning vector; IncP origin of replication; Tcr	Laboratory collection

### 2.3 Construction of *rsh* deletion mutant in SYK-6 and UT26

In-frame deletions of *rsh* gene in SYK-6 and UT26 were performed by employing the markerless gene deletion system for Sphingomonads (Kaczmarczyk et al., 2012; Lu and Huang, 2018). Primers used for gene deletion are listed in Supplementary Table S1. Briefly, flanking regions of *rsh*, approximately 500 bp up- and down-stream each, were amplified by PCR with the primer pairs syk6-5O/-5I and syk6-3O/-3I, then these two fragments were joined using overlap PCR with the primer pair syk6-5O/-3O. The resulting fragment was then cloned into the plasmid pAK405 via the BamHI/HindIII restriction site. Subsequently, the pAK405 derivative was delivered into the wild type strain via conjugal transfer from *E. coli* S17-1 (λ pir). After the conjugal transfer, bacteria were plated on LB media supplemented with kanamycin (100 μg/mL) and rifampicin (50 μg/mL). Individual colonies were re-streaked once on the same medium and then plated on LB media supplemented with 100 μg/mL streptomycin to select for the second homologous recombination event. The resulting colonies were re-streaked on both LB media supplemented with 100 μg/mL streptomycin and LB media containing 100 μg/mL kanamycin. Kanamycin-sensitive clones were analyzed by colony PCR using primers syk6-FO/-RO to confirm the successful deletion of *rsh*.

### 2.4 Measurement of growth curve

Bacterial strains were routinely grown in respective media at the shaking speed of 200 rpm, and their growth curves were drawn by measuring OD_600_ values at regular time intervals using a Spectrophotometer (Metash, Shanghai, China). All measurements were performed in triplicates.

### 2.5 Congo red binding assay

Congo red is an azo dye that can form complexes with different components in extracellular polymeric substances (EPS) (Semedo et al., 2015). The Congo red binding assay was carried out to evaluate bacterial EPS production (Lu and Huang, 2018).

Cultures were inoculated from a single colony and grown to an OD_600_ of 1 in LB broth; 10 μL of suspensions were spotted on LB plates containing 40 μg/mL Congo red. The plates were grown at 30°C to assess the morphology of colony. For quantitative analysis, the same number of cells from different culture conditions were suspended in 100 μg/mL Congo red in 0.9% saline and then incubated for 90 min with shaking at 30 rpm at room temperature. After incubation, the supernatant was obtained by centrifugation, and then the OD_490_ of the supernatant was measured as A_1_. The OD_490_ of Congo red solution without bacterial fluid was recorded as A_0_. All measurements were performed in triplicates. The percentage of Congo red stain was calculated to represent the abundance of EPS, by the formula:


$$\mathrm{Congo}\;\mathrm{red}\;\mathrm{stain}\;\left(\%\right)=\frac{A_{0}-A_{1}}{A_{0}}\times 100\%$$

### 2.6 AHL bio-assay and AHL profile identification by LC–MS/MS

The AHL production was analyzed either by cross-feeding assay or paper disk assay, according to our previous publication (Lu and Huang, 2018). Briefly, in the cross-feeding assay, the AHL reporter strain A136 and the tested strains were streaked side by side on the agar plates pre-covered with 50 μL *X*-gal (20 mg/mL stock solution in dimethylformamide). In the paper disk assay, ethyl acetate extracts of samples were spotted onto paper disks and incubated in LB soft agar plates mixed with A136 plus *X*-gal. The blue coloration results were recorded accordingly. LC–MS/MS was employed to identify the AHL profiles according to Huang et al. (2019).

### 2.7 Quantitative real time PCR (qRT-PCR)

qRT-PCR on *luxI/R* homologs was done as described previously (Lu and Huang, 2018). Briefly, cells of UT26 and SYK-6 at different growth phases and culture conditions were harvested and their total RNA was extracted with RNAprep pure Cell/Bacteria Kit (Tiangen Biotech, Beijing, China) according to the manufacturer’s instructions. cDNA was synthesized using FastKing gDNA Dispelling RT SuperMix (Tiangen Biotech, Beijing, China) and was used as the template for qRT-PCR amplification by using SYBR Premix Ex TaqTM Kit (Tli RNaseH Plus) (TaKaRa, Dalian, China) in Applied Biosystems StepOnePlus TM Real-Time PCR System (Thermo Scientific, Waltham, MA, United States). Primers were designed on the Sangon website^1^ and listed in Supplementary Table S2. The relative gene expression level of the mutant to the wild-type strain was analyzed using the 2^−∆∆Ct^ analysis method (Lu and Huang, 2018).

### 2.8 Detection of ppGpp

Bacterial cells at the early stationary phase were collected for ppGpp detection using the formic acid method (Lu and Huang, 2018). The presence of ppGpp in the extract was then determined by HPLC (Agilent, Santa Clara, CA, United States) analysis using a ZORBAX SB-C18 column (4.6 × 150 mm, 5 μm, Agilent). The mobile phase consisted of 125 mM KH_2_PO_4_, 10 mM tetrabutyl ammonium dihydrogen phosphate, 60 mL/L methanol, and 1 g/L KOH (pH 6.0). The column temperature was 40°C and the flow rate was 1.0 mL/min. The ppGpp was monitored at 254 nm and identified by comparison with the retention time of 100 μM ppGpp standards (TriLink Biosciences, San Diego, CA, United States). The ppGpp standard was eluted at around 71 min under the above-mentioned conditions to confirm the presence of ppGpp.

### 2.9 Statistical analyses

A Student’s *t*-test with a *p*-value cut-off of 0.05 was used to test significance of differences using the software SPSS (version 25).

## 3 Results

### 3.1 *In silico* survey for *luxI* homologs in sphingomonads

**Figure 1:**
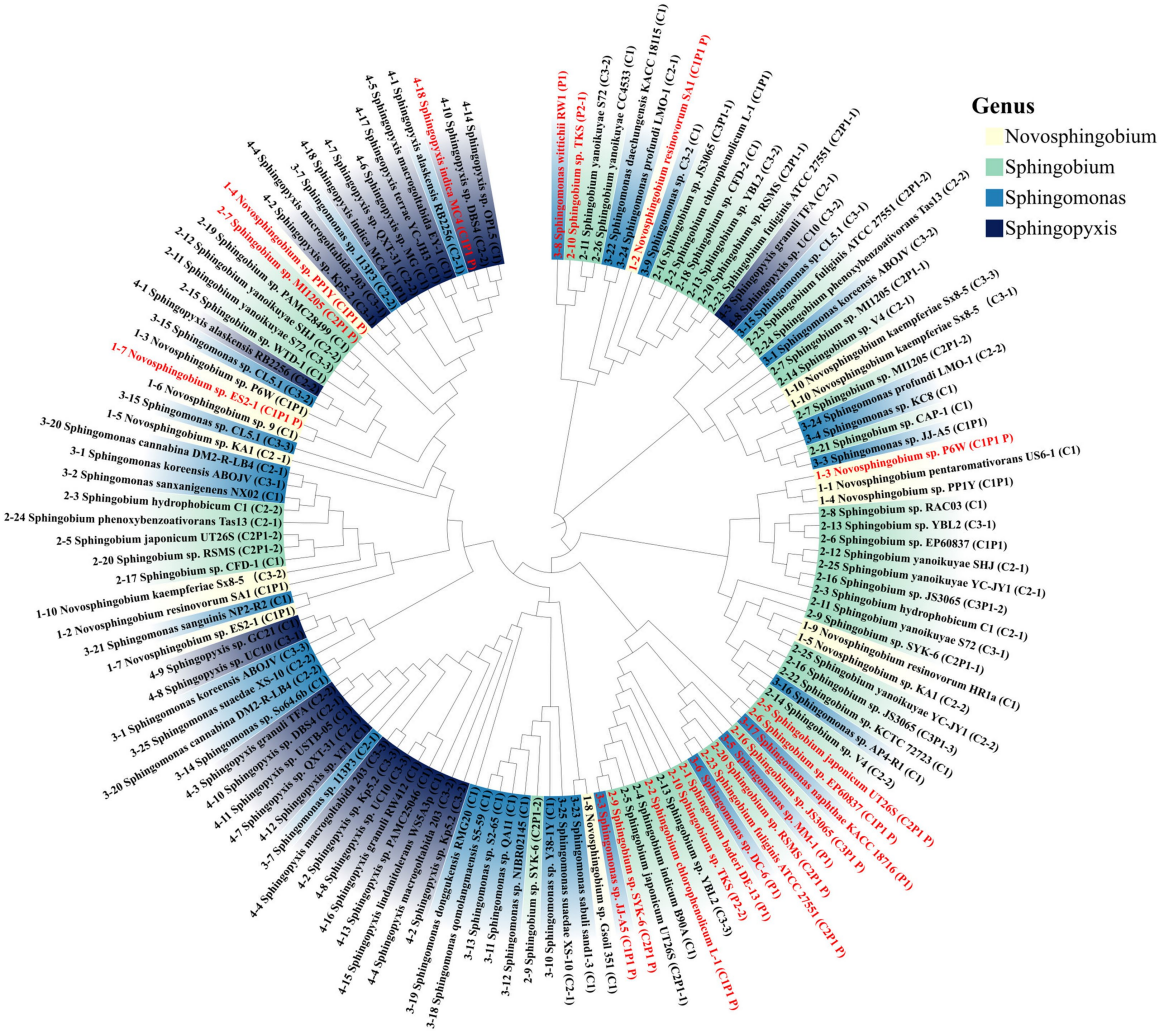
Phylogenetic tree of the LuxI-type synthases from Sphingomonads strains with completed genome sequence. In total, 131 sequences from 79 strains are plotted in the tree. The synthases located on the plasmid are depicted in red, and the synthases located on the chromosome are depicted in black. 59 out of 79 strains harbor *luxI* homologs in chromosome only, while 14 out of 79 strains harbor *luxI* homologs in both chromosome and plasmid. Different genera are highlighted in different colors. Each strain is assigned a serial number that depicted in front of the strain names. If a strain contains multiple *luxI* homologs then the total number and the location was indicated in brackets after the strain name. C for chromosome and P for plasmid. Different genes in the same strain are distinguished by a serial number or P. For example, (C2P1 P) means there are two *luxI* genes on the chromosome and one on the plasmid, this one is on the plasmid, the other two genes of this strain are indicated as (C2P1–1) and (C2P1–2).

Up to November 2023, 158 strains of sphingomonads had their whole genome sequenced. Among them, 79 strains were found to harbor *luxI* homologs, including 10 *Novosphingobium* spp., 18 *Sphingopyxis* spp., 26 *Sphingobium* spp., and 25 *Sphingomonas* spp. (Supplementary Table S3). The number of *luxI* homologs in each strain ranged from 1 to 4. 36 out of 79 strains contained more than one *luxI* homolog, and 13 out of 79 strains contained 3 *luxI* homologs, such as *Sphingobium japonicum* UT26 and *Sphingobium* sp. SYK-6 (Figure 1).

The majority of these *luxI* homologs (110 out of 131) were located on chromosome. More than two thirds, 59 out of 79, of these strains harbored *luxI* homologs in chromosome only, while 14 out of 79 strains harbored *luxI* homologs in both chromosome and plasmid (Figure 1). The phylogenetic tree showed that these *luxI* homologs in sphingomonads were not phylogenetically related (Figure 1). *LuxI* homologs from different genera mingled with each other and even *luxI* homologs from the same strain rarely clustered together.

### 3.2 The physical locations of *rsh* and *luxI/R* homologs in UT26 and SYK-6

**Figure 2:**
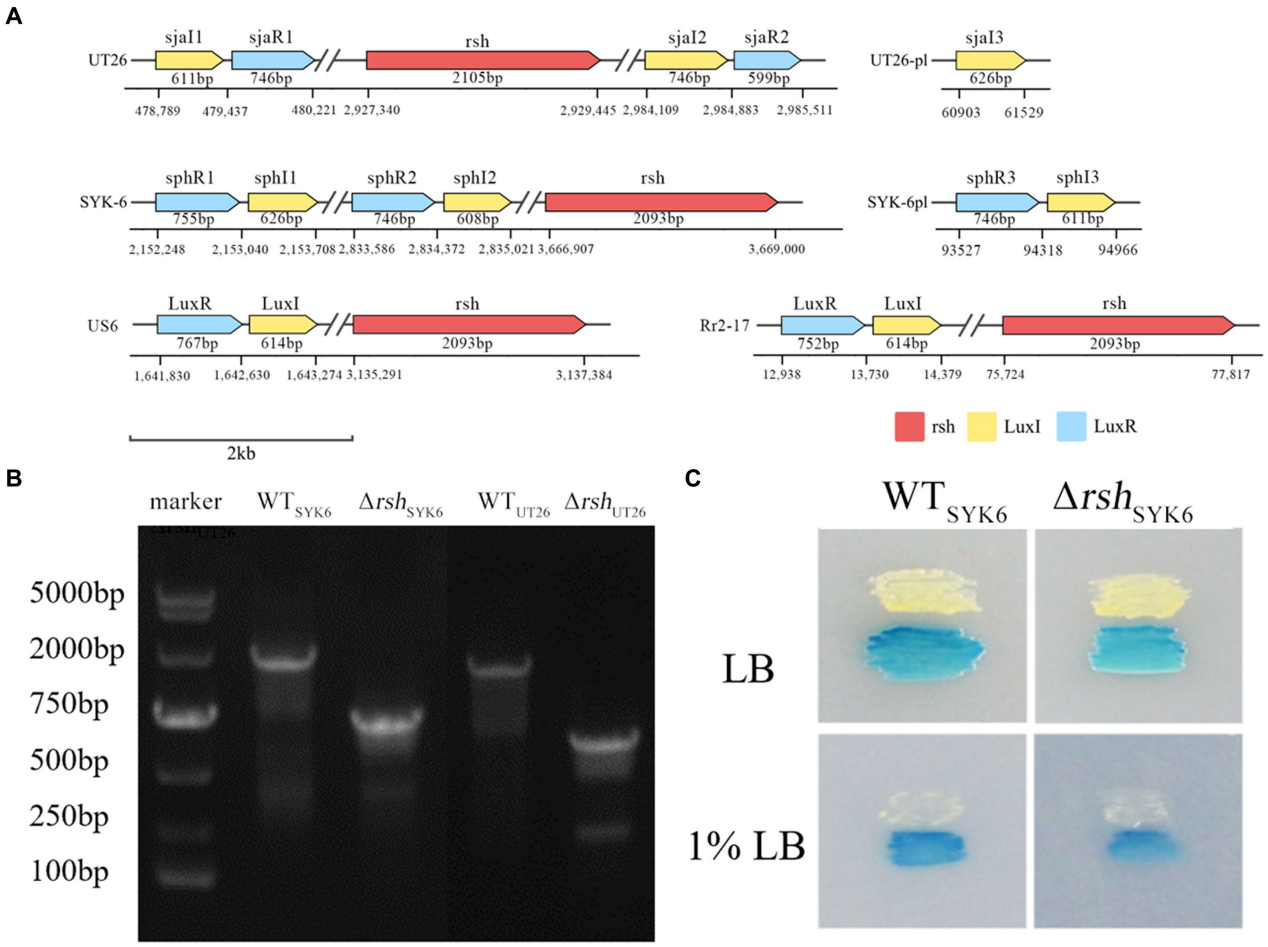
**(A)** Schematic diagram of the gene organization and location of *rsh* and *luxI/R* homologs in the genome of UT26, SYK-6, US6 and Rr2-17. **(B)** Agarose gel electrophoresis of PCR products from the wild type and the mutant using the same primer set. The shorter PCR products from the mutants indicates successful deletion of rsh gene. **(C)** Cross feeding assay for AHL production by the WT_UT26_ and Δrsh_UT26_ on LB and 1% LB agar plates. The blue coloration of the indicator strain A136 indicates the positive AHL production.

Sequence analysis on genomes of UT26 and SYK-6 indicated that both UT26 and SYK-6 contained two AHL synthase genes on the chromosome and one on a plasmid. These genes were named *sjaI* 1–3 for UT26 and *sphI* 1–3 for SYK-6. Except for *sjaI*3, other *luxI* homologs had corresponding *luxR* homologs in their close vicinity. On the other hand, one *rsh* gene around 2 kb was found on the chromosome of either UT26 or SYK-6. The detailed sequence information and location of *rsh* and these QS genes were plotted in Figure 2A, with reference strains US6-1 and Rr-2-17.

In UT26, both *sjaI/R*1 and *sjaI/R*2 located on the chromosome, and the solo *sjaI3* located on the plasmid. The *sjaI/R*1 located distantly upstream to *rsh*, around 2.5 million bases apart, while the *sjaI/R*2 located closely downstream to *rsh*, around 50 kb apart. Whereas in SYK-6, both *sphI/R*1 and *sphI/R*2 located distantly upstream to *rsh*, around 1.5 and 0.8 million bases apart, respectively. The *sphI/R*3 located on the plasmid. For the reference strains US6-1 and Rr2-17, the distances between *rsh* and the *luxI/R* homologs were 1.5 million bases and 60 kb apart, respectively. The physical distance between the *rsh* and QS genes on the chromosome were rather random among species.

### 3.3 Deletion of *rsh* and AHL production in UT26 and SYK-6

Successful deletion of the *rsh* gene in UT26 and SYK-6 was confirmed by PCR amplification of the sequence around the *rsh* gene. PCR products from both mutants were about 2 kb less than those of their wild type strains, due to the deletion of the *rsh* gene fragment (Figure 2B). The mutant was deficient in ppGpp production compared to the wild type strain, further proved the successful deletion of *rsh* (Supplementary Figure S1).

LC–MS/MS analysis indicated that both Δ*rsh*_UT26_ and Δ*rsh*_SYK-6_ produced AHL profiles similar to their wild type strains in rich LB medium, while in the minimal medium 1% LB, Δ*rsh*_SYK-6_ almost lost AHLs production (Table 2). Strain UT26 mainly produced C12-HSL, with a small amount of C14-HSL and 3-OH-C8-HSL. Whereas SYK-6 produced C14-HSL, 3-OH-C8-HSL, 3-OH-C6-HSL, and a trace amount of C8-HSL when grown in LB media (Supplementary Figure S2). The AHL signals from WT_SYK-6_ and Δ*rsh*_SYK-6_ were confirmed by the reporter strain A136, and both triggered a blue coloration in A136, indicating a positive AHL production in both strains (Figure 2C). These results indicated that deletion of *rsh* did not abolish the AHL production, but did affect their AHL profiles in both strains.

**Table 2:** Identification of AHL profiles in the wild type and mutant of UT26 and SYK-6.

Strain	WT_UT26_	Δ*rsh*_UT26_	WT_SYK-6_	Δ*rsh*_SYK-6_	WT_SYK-6_	Δ*rsh*_SYK-6_
Medium	1/3LB		LB		1%LB	
AHLs profile	C12-HSL C14-HSL (ta) 3-OH-C8-HSL (ta)	C12-HSL C14-HSL (ta) 3-OH-C8-HSL (ta)	C8-HSL (ta) C14-HSL 3-OH-C6-HSL 3-OH-C8-HSL	C14-HSL 3-OH-C6-HSL 3-OH-C8-HSL	C14-HSL 3-OH-C8-HSL	3-OH-C8-HSL (ta)

Ta in brackets means trace amount.

### 3.4 Regulatory effects of *rsh* on QS related activities in UT26

**Figure 3:**
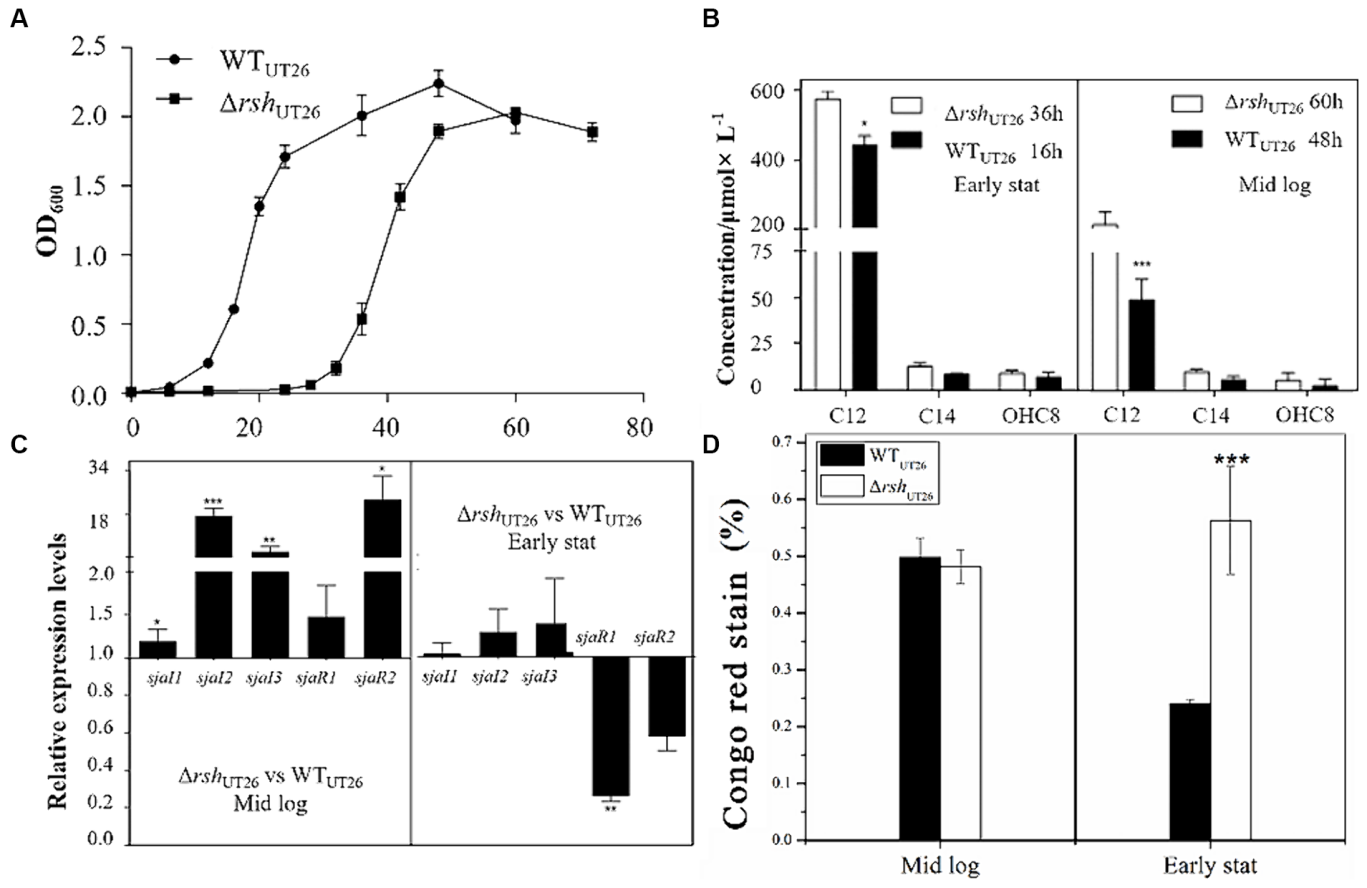
The growth curves, quorum sensing gene expression, AHL production and EPS production by UT26 in the 1/3 LB media. **(A)** Growth curves of WT_UT26_ and Δrsh_UT26_ under the shaking culture condition in 1/3 LB broth. **(B)** Concentration of the AHLs identified by LC–MS/MS in the WT_UT26_ and Δrsh_UT26_ in the middle log phase and the early stationary phase, respectively. **(C)** Relative expression levels of *luxI/R* homologs in the Δrsh_UT26_ compared to the WT_UT26_ in the middle log phase and the early stationary phase. **(D)** The Congo red removed percentage as the indicator of the EPS production for WT_UT26_ and Δrsh_UT26_ in the middle log phase and the early stationary phase. “^*^” represents *p* < 0.05; “^**^” represents *p* < 0.01; “^***^” represents *p* < 0.001.

Growth curves of Δ*rsh*_UT26_ and WT_UT26_ indicated that deletion of *rsh* rendered a delay in cell proliferation, but their overall biomasses in individual growth phase were similar (Figure 3A). The AHL production analysis indicated that in the middle log phase, AHL productions of Δ*rsh*_UT26_ and WT_UT26_ were similar, while in early stationary phase, Δ*rsh*_UT26_ remained high production of C12-HSL, which was significantly higher than WT_UT26_ (Figure 3B).

Relative gene expressions of *sjaI*1-3 and *sjaR*1-2 in Δ*rsh*_UT26_ compared to WT_UT26_ were measured (Figure 3C), results indicated that in the middle log phase, all these five genes were upregulated in Δ*rsh*_UT26_, especially *sjaI*2-3 and *sjaR*2. Whereas in the early stationary phase, *sjaI*1-3 was slightly upregulated, but *sjaR*1-2 was significantly downregulated. The consistent upregulated expression of these *sjaIs* in Δ*rsh*_UT26_ explained the higher production of AHLs in the mutant. The Δ*rsh*_UT26_ and WT_UT26_ showed similar EPS production in the middle log phase. However, in the early stationary phase, the mutant showed much higher EPS production than the wild type (Figure 3D).

Overall, these results indicated that for strain UT26, *rsh* negatively regulated the expression of most QS genes, which in turn negatively regulated AHLs and EPS production. These regulatory effects were growth phase dependent.

### 3.5 Regulatory effects of *rsh* on QS related activities in SYK-6

Since *rsh* gene was believed to function when nutrition was limited, therefore, 1% LB medium was used as a poor medium to mimic a nutrient-limited culture condition. Regulatory effects of *rsh* on AHL/EPS production and gene expression were tested in both rich media (LB) and poor medium (1% LB).

**Figure 4:**
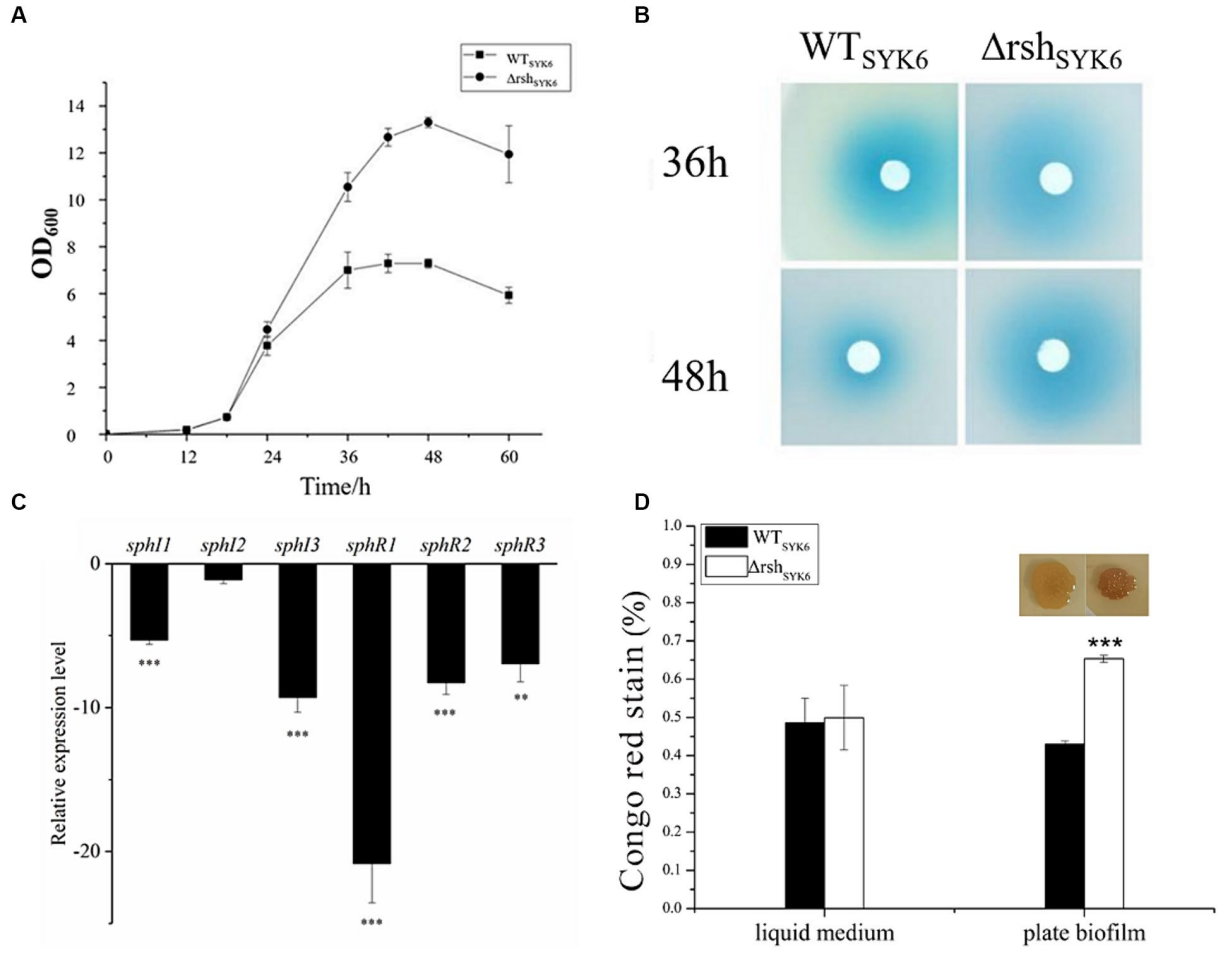
The growth curves, AHL production assay, quorum sensing gene expression assay and EPS production assay by SYK-6 in the LB media. **(A)** Growth curve of WT_SYK-6_ and Δrsh_SYK-6_ under the shaking culture condition in LB broth. **(B)** Disk diffusion assay for AHL production in the WT_SYK6_ and the Δrsh_SYK-6_ grown in the LB broth. The blue coloration indicates the presence of AHL. **(C)** Relative expression levels of *luxI/R* homologs in the Δrsh_SYK-6_ compared to the WT_SYK-6_. **(D)** Congo red assay for EPS production of the WT_SYK-6_ and the Δrsh_SYK-6_ grown in the LB broth or plates. Colony morphology of strains on the LB plates containing 40 μg/mL Congo red. Colony of Δrsh_SYK-6_ was redder than the wild type. One representative experiment out of three independent biological replicates is shown. “^*^” represents *p* < 0.05; “^**^” represents *p* < 0.01; “^***^” represents *p* < 0.001.

In LB medium, growth curves indicated that deletion of *rsh* significantly promoted the biomass of Δ*rsh*_SYK-6_ at stationary phase (Figure 4A). AHL production at 36 and 48 h were visualized. Δ*rsh*_SYK-6_ showed robust AHL production, the signals were stronger than WT_SYK-6_ at 48 h (Figure 4B). However, relative expressions of most QS genes were significantly downregulated in Δ*rsh*_SYK-6_, especially for *sphI*3 and *sphR*1. Notably, expression of *sphI* 2 was not significantly affected (Figure 4C). EPS production of Δ*rsh*_SYK-6_ was similar to that of the wild type when grown in the liquid medium, while was significantly higher than WT_SYK-6_ when grown in plate biofilm (Figure 4D). These results indicated that deletion of *rsh* downregulated most QS genes, the higher AHL and EPS production in the mutant might be attributed to the consistent expression of *sphI*2, and the higher biomass.

**Figure 5:**
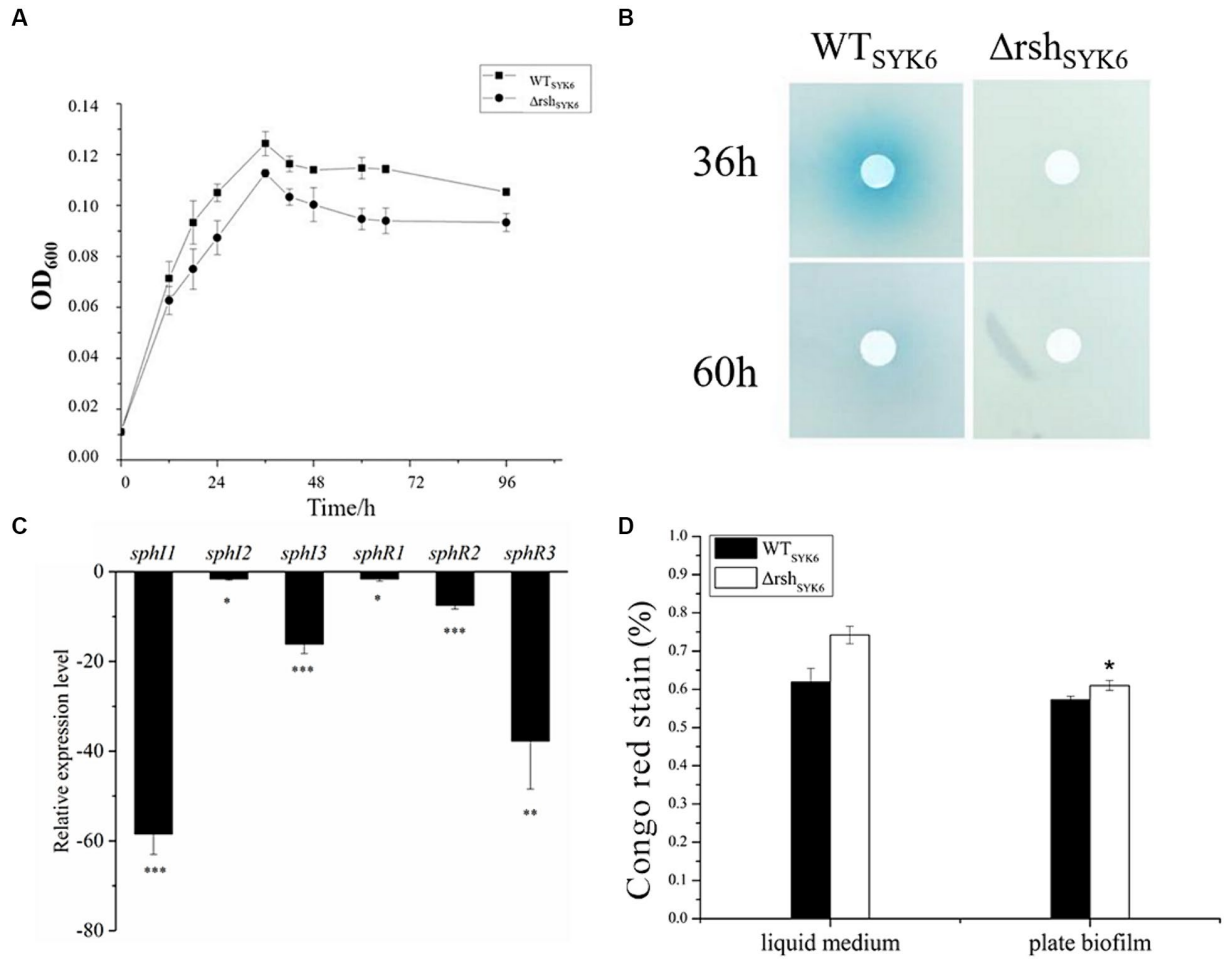
The growth curves, AHL production assay, quorum sensing gene expression assay and EPS production assay by SYK-6 in the 1% LB media. **(A)** Growth curve of WT_SYK-6_ and Δrsh_SYK-6_ under the shaking culture condition in 1%LB broth. **(B)** Disk diffusion assay for AHL production in the WT_SYK-6_ and the Δrsh_SYK-6_ grown in the 1% LB broth. The blue coloration indicates the presence of AHL. **(C)** Relative expression levels of *luxI/R* homologs in the Δrsh_SYK-6_ compared to the WT_SYK-6_ in 1%LB broth. **(D)** Congo red assay for EPS production of the WT_SYK-6_ and the Δrsh_SYK-6_ grown in the 1% LB broth or plates. “^*^” represents *p* < 0.05; “^**^” represents *p* < 0.01; “^***^” represents *p* < 0.001.

In 1% LB broth medium, deletion of *rsh* did not affect the growth of SYK-6 significantly (Figure 5A). Meanwhile, the mutant did not show AHL signals in broth extract (Figure 5B). LC–MS/MS analysis indicated that only 3-OH-C8-HSL, C14-HSL were produced by the WT_SYK-6_ in 1%LB, while only trace amount of 3-OH-C8-HSL was detected in Δ*rsh*_SYK-6_ in 1% LB (Table 2). This was contrary to the cross-feeding assay that on 1% LB agar plates, Δ*rsh*_SYK-6_ triggered a strong blue coloration of the reporter strain A136 just like the WT_SYK-6_ (Figure 2C). Gene expression analysis indicated that most QS genes were downregulated in the mutant, especially *sphI*1 and *sphR*3. Notably, *sphI*2 was the least affected gene (Figure 5C). The downregulated pattern of *rsh* on QS genes was different from what was found in the LB medium (Figure 4C). On the other hand, the EPS production of the mutant was always higher than that of the wild type in both broth and plate cultivation (Figure 5D).

Indeed, the regulatory effect of *rsh* on the QS system in SYK-6 was affected by nutrient availability and culture conditions. Specifically, planktonic cultivation in broth or attached growth on agar plate (biofilm) affected the regulatory effect of *rsh* on QS system.

## 4 Discussion

Results of this study demonstrated that the deletion of *rsh* did not abolish AHL productions in UT26 and SYK-6, it even promoted the production of some individual AHL molecules, in other words, *rsh* showed a negative regulatory effect on AHL production in UT26 and SYK-6. This was contrary to the positive regulatory effect of *rsh* on AHL production in US6-1 and Rr 2–27 (Gan et al., 2009; Lu and Huang, 2018). The basic reason for this discrepancy is due to the multiple QS circuits in UT26 and SYK-6. Although it has been reported that in *P. aeruginosa*, different class of QS systems, the AHL-based and quinolone signal-based QS circuits reacted to stringent response differently (Schafhauser et al., 2014), our study provided more sophisticated knowledge that QS circuits belong to the same class of QS system might react to stringent response differently.

In UT26, higher production of C12-HSL in Δ*rsh*_SYK-6_ was consistent with the upregulated expression of most QS genes in the mutant strain. While in SYK-6, although some QS genes were downregulated, the mutant still produced AHLs no less than the wild type. Notably, expressions of *sjaI*1 in UT26 and *sphI*2 in SYK-6 were not very significantly affected by *rsh* deletion. Whereas both the AHL synthase genes on plasmids were sensitive to *rsh* deletion. Combined with the distance and location information of these genes, we deduced that the effect of *rsh* on QS gene expression was not distance related, but the location, on the chromosome or plasmid, did matter (Figure 2A). In our previous study on *Ensifer* spp., QS genes on plasmid were very different from those in the chromosome in response to changing environmental conditions (Zeng et al., 2017). The genetic base for the regulatory mechanisms of *rsh* on QS genes requires more study.

According to growth curves, the deletion of *rsh* affected the growth of UT26 and SYK-6 in different ways. In UT26, Δ*rsh*_UT26_ displayed a longer lag phase than WT_UT26_, but eventually, their biomasses in stationary phases were similar. While in SYK-6, growth phases of WT_SYK-6_ and Δ*rsh*_SYK-6_ proceeded at a similar pace, but Δ*rsh*_SYK-6_ showed remarkably higher biomass than WT_SYK-6_ in the stationary phase, only in rich medium. Platitudinous nutrients and a lack of stringent response to slow down the growth rate in the stationary phase may explain this phenomenon. One important function of the stringent response is to slow down the growth rate when nutrients become limited (Fritsch et al., 2020). This result also reminds us that, even in the same genus, stringent responses may function differently in different species.

Biofilm formation and EPS production are two physiological activities that are closely related to QS (Sperandio et al., 2001; Tan et al., 2014). In SYK-6, the fact that Δ*rsh*_SYK-6_ produced detectable AHLs on 1% LB plate (Figure 4B) but not in 1% LB broth (Figure 5B) indicated that biofilm condition affected the execution of *rsh* regulatory outcome on QS. EPS production in mutants was generally higher than that of the wild types in both strains, which was consistent with the higher QS signal production. All these results suggested that QS and stringent response formed complex regulatory networks, which were affected by bacterial culture and growth conditions.

The intricate regulatory networks may guarantee delicate metabolism regulation that helps sphingomonads to survive in a broad range of econiches. Moreover, Sphingomonads contain lots of strains that have promising remediation potential for a variety of organic pollutants, such as polycyclic aromatic hydrocarbons (PAHs), dioxins, and chlorinated phenols, etc. (Waigi et al., 2017; Samantarrai et al., 2020; Jiang et al., 2022). A better understanding of the regulatory mechanisms and networks in sphingomonads will certainly help to utilize their metabolic functions in environmental bioremediation. In our previous studies, we found that QS in *Novosphingobium pentaromativorans* US6-1 affected the PAHs degradation ability by altering cell surface properties, while the *rsh* gene in US6-1 played multiple roles in its accommodation to different environmental pollutants (Chen and Huang, 2020; Lu et al., 2023).

In conclusion, this study provided novel data that the regulatory effect of stringent response on QS activities in sphingomonads was rather complicated; it was species-specific, and culture condition and growth phase-dependent. Unlike the positive regulatory effect that had been reported for US6-1 and Rr 2–17 with a single *luxI* homolog, our study on UT26 and SYK-6 with multiple *luxI* homologs showed a negative regulatory effect of *rsh* on QS-related bioactivities, including QS gene expression, QS signal production, and EPS production. Our study provides complementary information to our current knowledge, which is essential to understand the intricate network between QS and stringent response.

## Data availability statement

The datasets presented in this study can be found in online repositories. The names of the repository/repositories and accession number(s) can be found in the article/Supplementary material.

## Author contributions

YX: Data curation, Formal analysis, Investigation, Methodology, Software, Visualization, Writing – original draft, Writing – review & editing. XC: Data curation, Formal analysis, Investigation, Visualization, Writing – original draft, Writing – review & editing. HL: Conceptualization, Data curation, Formal analysis, Investigation, Methodology, Software, Visualization, Writing – original draft, Writing – review & editing. TJ: Data curation, Formal analysis, Investigation, Methodology, Software, Validation, Visualization, Writing – original draft, Writing – review & editing. YW: Data curation, Validation, Visualization, Writing – original draft. LL: Data curation, Investigation, Methodology, Resources, Validation, Writing – original draft, Writing – review & editing. SD: Writing – review & editing, Conceptualization, Data curation, Formal analysis, Investigation, Supervision, Writing – original draft. YH: Writing – review & editing, Conceptualization, Data curation, Formal analysis, Funding acquisition, Investigation, Methodology, Project administration, Resources, Software, Supervision, Validation, Visualization, Writing – original draft.

## Footnotes

^1^ http://www.sangon.com/
