# Supplementary material for: Regulation of quorum sensing activities by the stringent response gene rsh in sphingomonads is species-specific and culture condition dependent
Source: Front Microbiol. 2024 Apr 4;15:1368499. doi: 10.3389/fmicb.2024.1368499 (PMC11024222; doi:10.3389/fmicb.2024.1368499)
Supplement: Supplementary file 1 [file Table_1.DOCX]

**Supplementary tables and Figures**

**Figure S1** Measurement of ppGpp by HPLC. ppGpp peaked at 71min. In WT_UT26_ there was a peak at 71min while the peak did not appear inΔrsh_UT26_, indicating that ppGpp was no longer produced after the deletion of *rsh* in UT26.

**Figure S2** LC-MS/MS identification of AHLs profiles produced by WT_SYK6_ (a) and Δrsh_SYK6_ (b) grown in LB medium. (c) LC-MS/MS spectrograms of standard AHLs. (d) MS spectrograms of four AHLs, 3-OH-C6-HSL, 3-OH-C8-HSL, C8-HSL and C14-HSL.

**Figure S1**


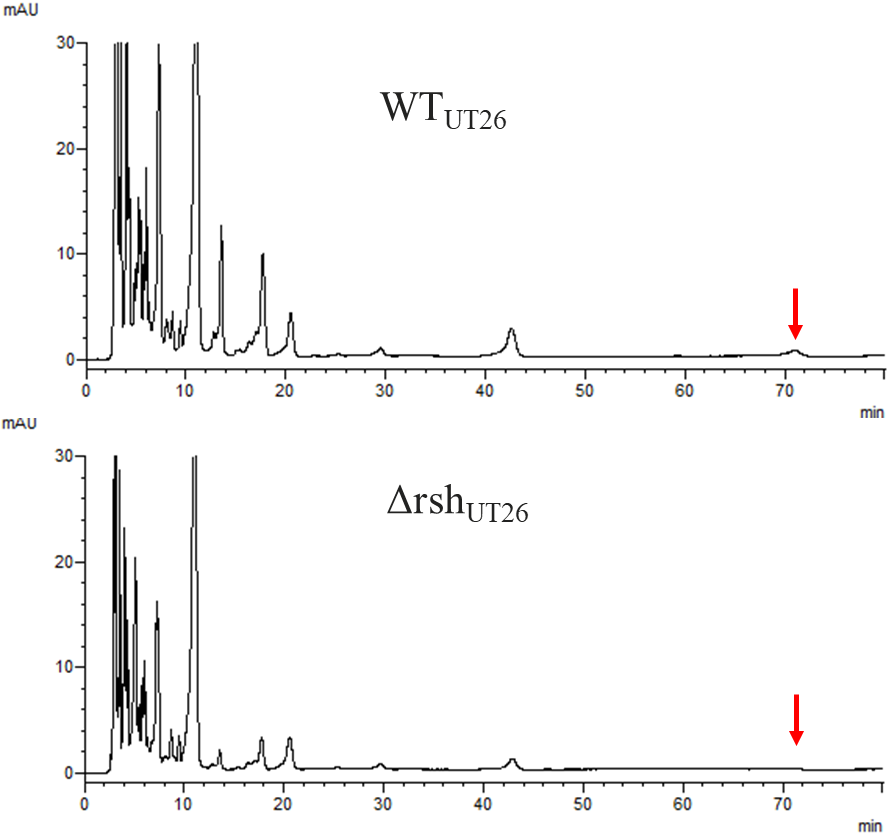


**Figure S2**


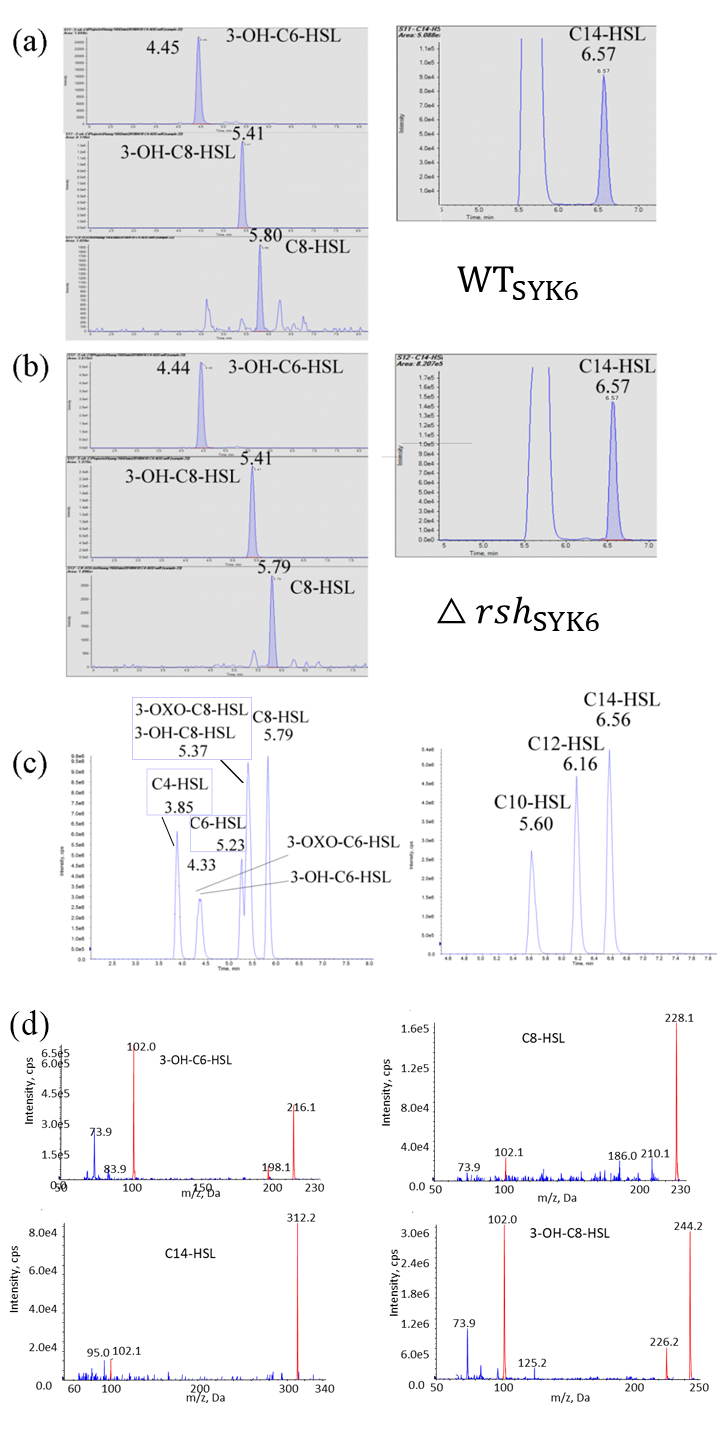


**Table S1 Primers used for mutant construction.**

| **primers** | **Primers sequence(5’→3’)** | **Application** |
| --- | --- | --- |
| Syk6-5O | CGCAAGCTTGCACGAGGAAGGAAATGG | *rsh* deletion for  SYK-6 |
| Syk6-5I | tgtttaaacttagtggatgggTTGGCGCAACATCCCGTC |  |
| Syk6-3O | CGCTCTAGACGCCGGTCGCATAGATGA |  |
| Syk6-3I | cccatccactaagtttaaacaGAGCGGGTCTGACCGGAC |  |
| Syk6-FO | GCAGCGTCAGCGAATAGCC |  |
| Syk6-RO | CCGGACTTACCCGCAAAA |  |
| UT26-FO | AGCGTCACCGAATAGCCC | *rsh* deletion for UT26 |
| UT26-5O | CGCAAGCTTTCCCACGGACTGGAGGT |  |
| UT26-5I | tgtttaaacttagtggatgggCTGGCGTAGCATCCTGTT |  |
| UT26-3I | cccatccactaagtttaaacaGACCGGGTATAAGCATGG |  |
| UT26-3O | CGCTCTAGACTGGCCCGTATCCCTCAA |  |
| UT26-RO | CGATCCATGCCTTTACTCCA |  |

**Table S2 Primers used for qRT-PCR.**

| Primer | Primer sequences（5’-3’） | Amplification target |
| --- | --- | --- |
| sphI1-86F | TCGATCTGTTGGGTTGGGAC | *sphI1* |
| sphI1-249R | GATATTGCCCAGGACATGCG |  |
| sphI2-159F | CTACCTGATCCTCACCGACG | *sphI2* |
| sphI2-309R | GAACCGGGTGATCTCGAGAA |  |
| sphI3-73F | CGGGTCTTCGTCGATCTTCT | *sphI3* |
| sphI3-263R | TCGCAAAGATCCGGGAAGAT |  |
| sphR1-31F | ATTGAGGCCTTTTACGACGC | *sphR1* |
| sphR1-211R | CATGGAAGACTTCGCTGAGC |  |
| sphR2-196F | GACTATTTTGACGCGCACCA | *sphR2* |
| sphR2-418R | CAACGGCGAAAGAACAGGAG |  |
| sphR3-289F | TCGATCATCTCTCTGACGGC | *sphR3* |
| sphR3-513R | GTGGATCCGGGAAATCTTGC |  |
| SYK6-16S-604F | TTGGAGAGGCGAGTGGAATT | 16S rRNA gene in sykSYK-6 |
| SYK6-16S-796R | CGAATTCCATGAACCCGGAC |  |
|  |  |  |
| TI1f | TGCACATCGTGAAAGAACCG | *sjaI1* |
| TI1r | AGGAGATCGACGAAGACGGA |  |
| TI2f | CGGAACTGCAAAAGATCGCC | *sjaI2* |
| TI2r | GAGTTGTCCCGAATCTGCCA |  |
| TI3f | TCAAGATCGACATCGGCTCG | *sjaI3* |
| TI3r | GGCCTGTACCGCAAAGATGA |  |
| TR1f | AGAGCAGCTGGGCTTTCAAT | *sjaR1* |
| TR1r | TAGAAATCCGCCCAACGGTC |  |
| TR2f | AGATGATCGACTGAACCGCC | *sjaR2* |
| TR2r | CTATCCGCAAGACTGGGTGG |  |
| T16Sf | CATCCCTATCGCGGTTTCCA | 16S rRNA gene in UT26 |
| T16Sr | CCCAACATCTCACGACACGA |  |

**Table S3: Summary of the location and number of the *luxI* homolog in genomes of Sphingomonads. C: Chromosome; P: Plasmid. Strains UT26 and SYK-6 were highlighted in red.**

| **Genus** | **Number** | **Species** | **Location and number of *luxI* homologs** |
| --- | --- | --- | --- |
| *Novosphingobium* | 1-1 | *pentaromativorans* US6-1 | C1 |
|  | 1-2 | *resinovorum* SA1 | C1P1 |
|  | 1-3 | sp. P6W | C1P1 |
|  | 1-4 | sp. PP1Y | C1P1 |
|  | 1-5 | sp.KA1 | C2 |
|  | 1-6 | sp. 9 | C1 |
|  | 1-7 | sp. ES2-1 | C1P1 |
|  | 1-8 | sp. Gsoil 351 | C1 |
|  | 1-9 | *resinovorum* HR1a | C1 |
|  | 1-10 | *kaempferiae* Sx8-5 | C3 |
| *Sphingobium* | 2-1 | *Baderi* DE-13 | P1 |
|  | 2-2 | *Chlorophenolicum* L-1 | C1P1 |
|  | 2-3 | *hydrophobicum* C1 | C2 |
|  | 2-4 | *indicum* B90A | C1 |
|  | 2-5 | *japonicum* UT26 | C2P1 |
|  | 2-6 | sp. EP60837 | C1P1 |
|  | 2-7 | sp. MI1205 | C2P1 |
|  | 2-8 | sp. RAC03 | C1 |
|  | 2-9 | sp. SYK-6 | C2P1 |
|  | 2-10 | sp. TKS | P2 |
|  | 2-11 | *yanoikuyae* S72 | C3 |
|  | 2-12 | *yanoikuyae* SHJ | C2 |
|  | 2-13 | [sp. YBL2](https://www.ncbi.nlm.nih.gov/datasets/taxonomy/484429/?utm_source=protein&utm_medium=referral&utm_campaign=KnownItemSensor:taxid) | C1 |
|  | 2-14 | [sp. V4](https://www.ncbi.nlm.nih.gov/datasets/taxonomy/3038927/) | C2 |
|  | 2-15 | sp. WTD-1 | C1 |
|  | 2-16 | sp. JS3065 | C3P1 |
|  | 2-17 | sp. CFD-1 | C1 |
|  | 2-18 | sp. CFD-2 | C1 |
|  | 2-19 | sp. PAMC28499 | C1 |
|  | 2-20 | sp. RSMS | C2P1 |
|  | 2-21 | sp. CAP-1 | C1 |
|  | 2-22 | sp. KCTC 72723 | C1 |
|  | 2-23 | *fuliginis* ATCC 27551 | C2P1 |
|  | 2-24 | *phenoxybenzoativorans Tas13* | C2 |
|  | 2-25 | *yanoikuyae YC-JY1* | C2 |
|  | 2-26 | *yanoikuyae CC4533* | C1 |
| *Spingomonas* | 3-1 | *koreensis* ABOJV | C3 |
|  | 3-2 | *sanxanigenens* NX02 | C1 |
|  | 3-3 | sp. JJ-A5 | C1P1 |
|  | 3-4 | sp. KC8 | C1 |
|  | 3-5 | sp. MM-1 | P1 |
|  | 3-6 | *wittichii* DC-6 | P1 |
|  | 3-7 | sp. 113P3 | C2 |
|  | 3-8 | *wittichii* RW1 | P1 |
|  | 3-9 | sp. C3-2 | C1 |
|  | 3-10 | sp. Y38-1Y | C1 |
|  | 3-11 | sp. QA11 | C1 |
|  | 3-12 | sp. NIBR02145 | C1 |
|  | 3-13 | sp. S2-65 | C1 |
|  | 3-14 | sp. So64.6b | C1 |
|  | 3-15 | sp. CL5.1 | C3 |
|  | 3-16 | sp. AP4-R1 | C1 |
|  | 3-17 | *naphthae* KACC 18716 | P1 |
|  | 3-18 | *qomolangmaensis* S5-59 | C1 |
|  | 3-19 | *donggukensis* RMG20 | C1 |
|  | 3-20 | *cannabina* DM2-R-LB4 | C2 |
|  | 3-21 | *sanguinis* NP2-R2 | C1 |
|  | 3-22 | *Daechungensis* KACC 18115 | C1 |
|  | 3-23 | *sabuli* sand1-3 | C1 |
|  | 3-24 | *profundi* LMO-1 | C2 |
|  | 3-25 | *suaedae* XS-10 | C2 |
| *Sphingopyxis* | 4-1 | *alaskensis* RB2256 | C2 |
|  | 4-2 | *fribergensis* Kp5.2 | C3 |
|  | 4-3 | *granuli* TFA | C2 |
|  | 4-4 | *macrogoltabida* 203 | C3 |
|  | 4-5 | *macrogoltabida* EY-1 | C1 |
|  | 4-6 | sp. MG | C1 |
|  | 4-7 | sp. QXT-31 | C2 |
|  | 4-8 | sp. UC10 | C3 |
|  | 4-9 | sp. GC21 | C1 |
|  | 4-10 | sp. DBS4 | C2 |
|  | 4-11 | sp. USTB-05 | C1 |
|  | 4-12 | sp. YF1 | C1 |
|  | 4-13 | sp. PAMC25046 | C1 |
|  | 4-14 | sp. OPL5 | C1 |
|  | 4-15 | *lindanitolerans* WS5A3p | C1 |
|  | 4-16 | *granuli* RW412 | C1 |
|  | 4-17 | *terrae* YC-JH3 | C1 |
|  | 4-18 | *indica* MC4 | C1P1 |
